# Supplementary material for: OpenMM 7: Rapid development of high performance algorithms for molecular dynamics
Source: PLoS Comput Biol. 2017 Jul 26;13(7):e1005659. doi: 10.1371/journal.pcbi.1005659 (PMC5549999; doi:10.1371/journal.pcbi.1005659)
Supplement: S1 Source Code — (ZIP) [file pcbi.1005659.s002.zip › OpenMM-7.0.1-Source/docs/api-c++/generated/OpenMM.CustomTorsionForce.html]

CustomTorsionForce — OpenMM @OPENMM\_MAJOR\_VERSION@.@OPENMM\_MINOR\_VERSION@ documentation


# CustomTorsionForce¶

*class* `OpenMM::``CustomTorsionForce`¶
:   This class implements interactions between sets of four particles that depend on the torsion angle between them. Unlike `PeriodicTorsionForce`, the functional form of the interaction is completely customizable, and may involve arbitrary algebraic expressions. In addition to the angle formed by the particles, it may depend on arbitrary global and per-torsion parameters.

    To use this class, create a `CustomTorsionForce` object, passing an algebraic expression to the constructor that defines the interaction energy between each set of particles. The expression may depend on theta, the torsion angle formed by the particles, as well as on any parameters you choose. Then call `addPerTorsionParameter()` to define per-torsion parameters, and `addGlobalParameter()` to define global parameters. The values of per-torsion parameters are specified as part of the system definition, while values of global parameters may be modified during a simulation by calling `Context::setParameter()`. Finally, call `addTorsion()` once for each torsion. After an torsion has been added, you can modify its parameters by calling `setTorsionParameters()`. This will have no effect on Contexts that already exist unless you call `updateParametersInContext()`.

    As an example, the following code creates a `CustomTorsionForce` that implements a harmonic potential:

    ```
    CustomTorsionForce* force = new CustomTorsionForce("0.5*k*(theta-theta0)^2");
    ```

    This force depends on two parameters: the spring constant k and equilibrium angle theta0. The following code defines these parameters:

    ```
    force->addPerTorsionParameter("k");
    force->addPerTorsionParameter("theta0");
    ```

    Expressions may involve the operators + (add), - (subtract), \* (multiply), / (divide), and ^ (power), and the following functions: sqrt, exp, log, sin, cos, sec, csc, tan, cot, asin, acos, atan, sinh, cosh, tanh, erf, erfc, min, max, abs, floor, ceil, step, delta, select. All trigonometric functions are defined in radians, and log is the natural logarithm. step(x) = 0 if x is less than 0, 1 otherwise. delta(x) = 1 if x is 0, 0 otherwise. select(x,y,z) = z if x = 0, y otherwise.

    Methods

    |  |  |
    | --- | --- |
    | `CustomTorsionForce` | Create a `CustomTorsionForce`. |
    | `getNumTorsions` | Get the number of torsions for which force field parameters have been defined. |
    | `getNumPerTorsionParameters` | Get the number of per-torsion parameters that the interaction depends on. |
    | `getNumGlobalParameters` | Get the number of global parameters that the interaction depends on. |
    | `getEnergyFunction` | Get the algebraic expression that gives the interaction energy for each torsion |
    | `setEnergyFunction` | Set the algebraic expression that gives the interaction energy for each torsion |
    | `addPerTorsionParameter` | Add a new per-torsion parameter that the interaction may depend on. |
    | `getPerTorsionParameterName` | Get the name of a per-torsion parameter. |
    | `setPerTorsionParameterName` | Set the name of a per-torsion parameter. |
    | `addGlobalParameter` | Add a new global parameter that the interaction may depend on. |
    | `getGlobalParameterName` | Get the name of a global parameter. |
    | `setGlobalParameterName` | Set the name of a global parameter. |
    | `getGlobalParameterDefaultValue` | Get the default value of a global parameter. |
    | `setGlobalParameterDefaultValue` | Set the default value of a global parameter. |
    | `addTorsion` | Add a torsion term to the force field. |
    | `getTorsionParameters` | Get the force field parameters for a torsion term. |
    | `setTorsionParameters` | Set the force field parameters for a torsion term. |
    | `updateParametersInContext` | Update the per-torsion parameters in a `Context` to match those stored in this `Force` object. |
    | `usesPeriodicBoundaryConditions` | Returns whether or not this force makes use of periodic boundary conditions. |

    `CustomTorsionForce`(*const* std::string &*energy*)¶
    :   Create a `CustomTorsionForce`.

        |  |  |
        | --- | --- |
        | Parameters: |  |

        - **energy** – an algebraic expression giving the interaction energy between three particles as a function of theta, the torsion angle between them

    int `getNumTorsions`() *const*¶
    :   Get the number of torsions for which force field parameters have been defined.

    int `getNumPerTorsionParameters`() *const*¶
    :   Get the number of per-torsion parameters that the interaction depends on.

    int `getNumGlobalParameters`() *const*¶
    :   Get the number of global parameters that the interaction depends on.

    *const* std::string &`getEnergyFunction`() *const*¶
    :   Get the algebraic expression that gives the interaction energy for each torsion

    void `setEnergyFunction`(*const* std::string &*energy*)¶
    :   Set the algebraic expression that gives the interaction energy for each torsion

    int `addPerTorsionParameter`(*const* std::string &*name*)¶
    :   Add a new per-torsion parameter that the interaction may depend on.

        |  |  |
        | --- | --- |
        | Parameters: |  |

        - **name** – the name of the parameter

        |  |  |
        | --- | --- |
        | Returns: | the index of the parameter that was added |

    *const* std::string &`getPerTorsionParameterName`(int *index*) *const*¶
    :   Get the name of a per-torsion parameter.

        |  |  |
        | --- | --- |
        | Parameters: |  |

        - **index** – the index of the parameter for which to get the name

        |  |  |
        | --- | --- |
        | Returns: | the parameter name |

    void `setPerTorsionParameterName`(int *index*, *const* std::string &*name*)¶
    :   Set the name of a per-torsion parameter.

        |  |  |
        | --- | --- |
        | Parameters: |  |

        - **index** – the index of the parameter for which to set the name
        - **name** – the name of the parameter

    int `addGlobalParameter`(*const* std::string &*name*, double *defaultValue*)¶
    :   Add a new global parameter that the interaction may depend on.

        |  |  |
        | --- | --- |
        | Parameters: |  |

        - **name** – the name of the parameter
        - **defaultValue** – the default value of the parameter

        |  |  |
        | --- | --- |
        | Returns: | the index of the parameter that was added |

    *const* std::string &`getGlobalParameterName`(int *index*) *const*¶
    :   Get the name of a global parameter.

        |  |  |
        | --- | --- |
        | Parameters: |  |

        - **index** – the index of the parameter for which to get the name

        |  |  |
        | --- | --- |
        | Returns: | the parameter name |

    void `setGlobalParameterName`(int *index*, *const* std::string &*name*)¶
    :   Set the name of a global parameter.

        |  |  |
        | --- | --- |
        | Parameters: |  |

        - **index** – the index of the parameter for which to set the name
        - **name** – the name of the parameter

    double `getGlobalParameterDefaultValue`(int *index*) *const*¶
    :   Get the default value of a global parameter.

        |  |  |
        | --- | --- |
        | Parameters: |  |

        - **index** – the index of the parameter for which to get the default value

        |  |  |
        | --- | --- |
        | Returns: | the parameter default value |

    void `setGlobalParameterDefaultValue`(int *index*, double *defaultValue*)¶
    :   Set the default value of a global parameter.

        |  |  |
        | --- | --- |
        | Parameters: |  |

        - **index** – the index of the parameter for which to set the default value
        - **defaultValue** – the default value of the parameter

    int `addTorsion`(int *particle1*, int *particle2*, int *particle3*, int *particle4*, *const* std::vector<double> &*parameters* = std::vector< double >())¶
    :   Add a torsion term to the force field.

        |  |  |
        | --- | --- |
        | Parameters: |  |

        - **particle1** – the index of the first particle connected by the torsion
        - **particle2** – the index of the second particle connected by the torsion
        - **particle3** – the index of the third particle connected by the torsion
        - **particle4** – the index of the fourth particle connected by the torsion
        - **parameters** – the list of parameters for the new torsion

        |  |  |
        | --- | --- |
        | Returns: | the index of the torsion that was added |

    void `getTorsionParameters`(int *index*, int &*particle1*, int &*particle2*, int &*particle3*, int &*particle4*, std::vector<double> &*parameters*) *const*¶
    :   Get the force field parameters for a torsion term.

        |  |  |
        | --- | --- |
        | Parameters: |  |

        - **index** – the index of the torsion for which to get parameters
        - **particle1** – [out] the index of the first particle connected by the torsion
        - **particle2** – [out] the index of the second particle connected by the torsion
        - **particle3** – [out] the index of the third particle connected by the torsion
        - **particle4** – [out] the index of the fourth particle connected by the torsion
        - **parameters** – [out] the list of parameters for the torsion

    void `setTorsionParameters`(int *index*, int *particle1*, int *particle2*, int *particle3*, int *particle4*, *const* std::vector<double> &*parameters* = std::vector< double >())¶
    :   Set the force field parameters for a torsion term.

        |  |  |
        | --- | --- |
        | Parameters: |  |

        - **index** – the index of the torsion for which to set parameters
        - **particle1** – the index of the first particle connected by the torsion
        - **particle2** – the index of the second particle connected by the torsion
        - **particle3** – the index of the third particle connected by the torsion
        - **particle4** – the index of the fourth particle connected by the torsion
        - **parameters** – the list of parameters for the torsion

    void `updateParametersInContext`(Context &*context*)¶
    :   Update the per-torsion parameters in a `Context` to match those stored in this `Force` object. This method provides an efficient method to update certain parameters in an existing `Context` without needing to reinitialize it. Simply call `setTorsionParameters()` to modify this object’s parameters, then call `updateParametersInContext()` to copy them over to the `Context`.

        This method has several limitations. The only information it updates is the values of per-torsion parameters. All other aspects of the `Force` (such as the energy function) are unaffected and can only be changed by reinitializing the `Context`. The set of particles involved in a torsion cannot be changed, nor can new torsions be added.

    bool `usesPeriodicBoundaryConditions`() *const*¶
    :   Returns whether or not this force makes use of periodic boundary conditions.

        |  |  |
        | --- | --- |
        | Returns: | true if force uses PBC and false otherwise |

High performance molecular simulation on GPUs


### Navigation

- Core Objects
- Forces
- Integrators
- Extras

©2015, Stanford University and the Authors.
|
Powered by Sphinx 1.3.1
& Alabaster 0.7.7
|
Page source
